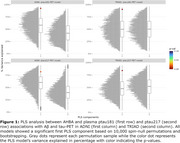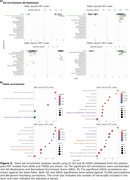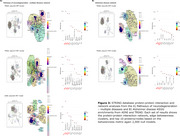# Mapping molecular pathways underlying the relationship between plasma ptau and Alzheimer’s disease pathology: an imaging‐transcriptomic study

**DOI:** 10.1002/alz70862_110121

**Published:** 2025-12-23

**Authors:** Min Su Kang, Julie Ottoy, Andrew Clappison, Gleb Bezgin, Thomas K Karikari, Gassan Massarweh, Jean‐Paul Soucy, Serge Gauthier, Mario Masellis, Andrew Lim, Walter Swardfager, Kaj Blennow, Henrik Zetterberg, Sandra E. Black, Pedro Rosa‐Neto, Maged Goubran

**Affiliations:** ^1^ Dr. Sandra E. Black Centre for Brain Resilience and Recovery, LC Campbell Cognitive Neurology, Hurvitz Brain Sciences Program, Sunnybrook Research Institute, University of Toronto, Toronto, ON Canada; ^2^ Artificial Intelligence and Computational Neurosciences Lab, Sunnybrook Research Institute, University of Toronto, Toronto, ON Canada; ^3^ Sunnybrook Research Institute, Toronto, ON Canada; ^4^ Hurvitz Brain Sciences Program, Sunnybrook Research Institute, Toronto, ON Canada; ^5^ University of Toronto Sunnybrook Research Institute, Toronto, ON Canada; ^6^ LC Campbell Cognitive Neurology Research Unit, Sunnybrook Research Institute, University of Toronto, Toronto, ON Canada; ^7^ Translational Neuroimaging Laboratory, The McGill University Research Centre for Studies in Aging, Montréal, QC Canada; ^8^ McGill University, Montreal, QC Canada; ^9^ Department of Psychiatry and Neurochemistry, Institute of Neuroscience and Physiology, The Sahlgrenska Academy, University of Gothenburg, Mölndal, Gothenburg Sweden; ^10^ University of Pittsburgh, Pittsburgh, PA USA; ^11^ McConnell Brain Imaging Centre ‐ McGill University, Montréal, QC Canada; ^12^ Montreal Neurological Institute, McGill University, Montreal, QC Canada; ^13^ Montreal Neurological Institute, McGill University, Montréal, QC Canada; ^14^ McConnell Brain Imaging Centre, Montreal Neurological Institute and Hospital, McGill University, Montreal, QC Canada; ^15^ Department of Neurology and Neurosurgery, and Department of Psychiatry, McGill Centre for Studies in Aging, McGill University, Montreal, QC Canada; ^16^ Division of Neurology, Department of Medicine, Sunnybrook Health Sciences Centre, Toronto, ON Canada; ^17^ Cognitive and Movement Disorders Clinic, Sunnybrook Health Sciences Center, Toronto, ON Canada; ^18^ University of Toronto, Toronto, ON Canada; ^19^ Dr. Sandra Black Centre for Brain Resilience & Recovery, Sunnybrook Research Institute, Toronto, ON Canada; ^20^ Hurvitz Brain Sciences Research Program, Sunnybrook Research Institute, Toronto, ON Canada; ^21^ Heart and Stroke Foundation Canadian Partnership for Stroke Recovery, Toronto, ON Canada; ^22^ Department of Psychiatry and Neurochemistry, Institute of Neuroscience and Physiology, The Sahlgrenska Academy, University of Gothenburg, Mölndal Sweden; ^23^ Clinical Neurochemistry Laboratory, Sahlgrenska University Hospital, Mölndal Sweden; ^24^ Clinical Neurochemistry Laboratory, Sahlgrenska University Hospital, Mölndal, Västra Götalands län Sweden; ^25^ Department of Neurodegenerative Disease, National Hospital for Neurology and Neurosurgery, UCL Institute of Neurology, London UK; ^26^ UK Dementia Research Institute at UCL, London UK; ^27^ Division of Neurology, Department of Medicine, University of Toronto, Toronto, ON Canada; ^28^ Dr. Sandra Black Centre for Brain Resilience & Recovery, Toronto, ON Canada; ^29^ Sunnybrook Research Institute, University of Toronto, Toronto, ON Canada; ^30^ McGill University Research Centre for Studies in Aging, Montreal, QC Canada; ^31^ McConnell Brain Imaging Centre, Montreal Neurological Institute, McGill University, Montreal, QC Canada; ^32^ Translational Neuroimaging Laboratory, Montreal, QC Canada; ^33^ Department of Medical Biophysics, University of Toronto, Toronto, ON Canada

## Abstract

**Background:**

Collective evidence suggests that plasma ptau181 and 217 reflect Alzheimer’s disease (AD) from the amyloid‐beta (Aβ) to tau pathologies. However, possible molecular pathways underlying the biological mechanisms that relate AD pathology with plasma ptau have not yet been elucidated.

**Method:**

We studied 549 participants from two cohorts: ADNI (Aβ‐: 83 CN; Aβ+: 171 CN, 97MCI, 39 AD; Aβ‐PET: [^18^F]AV45; tau‐PET: [^18^F]AV1451) and TRIAD (Aβ‐: 26 Young, 62 CN; Aβ+: 29 CN, 30 MCI, 24 AD; Aβ‐PET: [^18^F]AZD4694; tau‐PET: [^18^F]MK6240). Both cohorts included plasma ptau181 and 217, which were quantified using the SIMOA/Janssen. All images were processed using Freesurfer or PETsurfer into the DKT atlas. Linear regression models investigated the associations between plasma ptau and Aβ‐PET or tau‐PET, adjusted for age, sex, education, and APOEε4. Partial least squares (PLS) analysis identified a set of transcriptomic profiles from the Allen Human Brain Atlas (AHBA) associated with the ptau181‐PET (Aβ and tau‐PET) or ptau217‐PET relationships. Then, gene set enrichment analyses based on GO and KEGG databases and STRINGdb protein‐protein interactions were conducted to highlight which molecular/biological processes and cellular components are associated with the identified transcriptomic profiles.

**Result:**

The ptau181‐PET and ptau217‐PET relationships were significantly associated with the spatial distribution of the AHBA, explaining >90% and >82% in variance in the 1st PLS component from both cohorts, respectively (Figure 1). Subsequent gene enrichment analyses showed *mitochondrial metabolism* for ptau181‐PET and *synaptic function* for ptau217‐PET as converging GO AD‐Biodomains (Figure 2). The KEGG enrichment analyses identified *pathways of neurodegeneration* for ptau181‐PET and *cytokine‐cytokine receptor interaction* for ptau217‐PET as converging annotations (Figure 2). Notably, TRIAD also showed *Alzheimer’s disease* KEGG annotation from both ptau models (Figure 2). The STRINGdb analyses confirmed significant protein‐protein interactions and revealed MAPT, PINK1, and SNCA proteins with the largest betweenness metric within significant networks of KEGG terms from TRIAD, while BCL2L1, PINK1, and GSK3β proteins were identified in ADNI (Figure 3).

**Conclusion:**

Imaging‐transcriptomic analyses showed unique sets of transcriptomic profiles, highlighting mitochondrial metabolism and synaptic as key AD‐biodomains underlying the plasma ptau‐AD pathology relationship. Our study underscores the MAPT and SNCA proteins and intracellular signalling as salient molecular pathways in neurodegeneration and AD dementia.